# Comprehensive proteogenomic analysis of human embryonic and induced pluripotent stem cells

**DOI:** 10.1111/jcmm.14426

**Published:** 2019-06-25

**Authors:** Elvira Immacolata Parrotta, Stefania Scalise, Domenico Taverna, Maria Teresa De Angelis, Gianmarco Sarro, Marco Gaspari, Gianluca Santamaria, Giovanni Cuda

**Affiliations:** ^1^ Research Center for Advanced Biochemistry and Molecular Biology, Department of Experimental and Clinical Medicine University “Magna Graecia” of Catanzaro Catanzaro Italy

**Keywords:** human embryonic stem cells (hESCs), human induced pluripotent stem cells (hiPSCs), Ingenuity Pathway Analysis (IPA), mass spectrometry, pluripotency, post‐translational modifications, proteome, transcriptome

## Abstract

Although the concepts of somatic cell reprogramming and human‐induced pluripotent stem cells (hiPSCs) generation have undergone several analyses to validate the usefulness of these cells in research and clinic, it remains still controversial whether the hiPSCs are equivalent to human embryonic stem cells (hESCs), pointing to the need of further characterization for a more comprehensive understanding of pluripotency. Most of the experimental evidence comes from the transcriptome analysis, while a little is available on protein data, and even less is known about the post‐translational modifications. Here, we report a combined strategy of mass spectrometry and gene expression profiling for proteogenomic analysis of reprogrammed and embryonic stem cells. The data obtained through this integrated, multi‐“omics” approach indicate that a small, but still significant, number of distinct pathways is enriched in reprogrammed versus embryonic stem cells, supporting the view that pluripotency is an extremely complex, multifaceted phenomenon, with peculiarities that are characteristic of each cell type.

## 1 INTRODUCTION

Pluripotency is a property shared by certain types of cells that have the ability to differentiate, either spontaneously or following appropriate stimuli, into any of the three germ layers (ectoderm, mesoderm, and endoderm).^1^ Embryonic stem cells (ESCs) are the main example of pluripotent cells (PSCs) and represent an extraordinary tool for studying normal development, as well as for deciphering the molecular mechanisms underlying a number of complex diseases.^2^ Their use in laboratory, however, is accompanied by the need to overcome important ethical considerations that can be circumvented by reprogramming somatic cells into stem cells (termed induced pluripotent stem cells, iPSCs) through the forced expression of pluripotency‐promoting transcription factors.^3^ This strategy, developed by Takahashi and Yamanaka, represented a milestone in stem cell biology and paved the way for hundreds of studies related to this exciting field.^4^, ^5^, ^6^, ^7^, ^8^ Despite the numerous advances in stem cells research, the equivalence between human embryonic stem cells (hESCs) and human‐induced pluripotent stem cells (hiPSCs) still remains controversial. Different studies have shown quite a high similarity between embryonic and reprogrammed pluripotent stem cells,^9^ while others have instead found significant differences.^10^, ^11^ At the mRNA level, for example, early passage iPSCs show specific patterns of expression that are not detected in ESCs; this discrepancy seems to become less evident in late passage iPSCs.^12^ While the epigenomic and transcriptomic profiles of hiPSCs and hESCs have been widely discussed,^13^, ^14^, ^15^ less is instead known about proteomic patterns^16^ and post‐translational modifications,^17^ such as phosphorylation.^18^ Here, we have applied and integrated a multi‐‘omics’ strategy to dissect, at the transcriptional, translational and post‐translational level, two distinct human PSCs, the H9 embryonic stem cell line (hESCs), and the induced pluripotent stem cell line (hiPSC‐1), obtained by reprogramming of peripheral blood T‐lymphocytes from a healthy volunteer. Two additional hiPSC lines, one generated from peripheral blood T‐lymphocytes and one generated from skin fibroblasts (hiPSC‐2 and hiPSC‐3, respectively) were included in this study to validate the proteogenomic data.

## 2 MATERIAL AND METHODS

### 2.1 Generation and culture of induced pluripotent stem cells

A written informed consent for human blood and human skin fibroblasts collection was obtained from three healthy donors. The study was approved and supervised by the Ethics Committee of the ‘Magna Graecia’ University of Catanzaro and the research was carried out according to the World Medical Association Declaration of Helsinki. hiPSCs were generated from two different cell sources: T‐Lymphocytes isolated from peripheral blood mononuclear cells (PBMCs) (hiPSC‐1 and hiPSCs‐2 lines) and skin fibroblasts (hiPSCs‐3). Fibroblasts were isolated and expanded by the outgrowth method in DMEM containing 10% fetal bovine serum (FBS, Thermo Fisher Scientific) and 50 U/mL penicillin, and 50 µg/mL streptomycin. Cells were passaged twice before infection for iPSCs generation. Reprogramming of fibroblasts to pluripotency was carried out by non‐integrating Sendai‐virus‐mediated (CytoTune™‐iPS 2.0 Sendai Reprogramming Kit, Thermo Fisher Scientific) transfection of the four canonical transcription factors (*Oct4*, *Sox2*, *Klf4* and *c‐Myc*). 3 × 10^5^ fibroblasts were infected at a multiplicity of infection (MOI) of 5, yielding different iPSCs clones generated in feeder‐independent conditions on Matrigel‐coated dishes (BD Biosciences). For hiPSCs generation from T‐lymphocytes, PBMCs were cultured in AIM‐V medium (Thermo Fisher Scientific) containing 20% FBS, 50 U/mL penicillin, and 50 µg/mL streptomycin (Thermo Fisher Scientific), supplemented with 125 ng/mL Interleukine‐2 (IL‐2) (R&D Systems) and finally seeded onto CD3‐coated dishes (10 µg/mL, BD Biosciences) for T‐lymphocytes activation. 5 × 10^5^ T‐Lymphocytes were infected with Sendai virus (SeV)^19^ at MOI of 20 in feeder‐independent conditions. hiPSC lines (hiPSC‐1, hiPSC‐2, and hiPSC‐3) and hESCs H9 (the latter purchased from WiCell Research Institute) were cultured on Matrigel‐coated (BD Biosciences) dishes in mTeSR1 medium (STEMCELL Technologies) in a humidified incubator at 37°C at 5% CO_2_. Cells were passaged using Gentle Cell Dissociation Reagent (STEMCELL Technologies). All cell lines were tested for Mycoplasma before being used in experiments.

### 2.2 Assessment of pluripotency of generated iPSCs

Prior to pluripotency assessment, all generated hiPSCs lines were tested for SeV‐transgenes loss by reverse transcription polymerase chain reaction (RT‐PCR). Detection of SeV‐transgenes was performed in infected parental cells for presence, uninfected parental cells for absence, and generated hiPSCs for loss of viral transgenes. Pluripotency of generated hiPSCs was assessed for the expression of pluripotency‐associated genes (*OCT4*, *NANOG*, *SOX2*, *REX1* and *DNMT3B*) by quantitative real‐time PCR (qRT‐PCR) and for expression of pluripotency markers (NANOG and OCT4) by immunostaining. Additionally, generated hiPSCs were tested for markers of the three germ layers, NESTIN (ectoderm), BRACHYURY (mesoderm), and SOX17 (endoderm) on whole Embryoid Bodies (EBs) by immunostaining and by qRT‐PCR for endoderm (*SOX7* and *SOX17*), mesoderm (*HAND1, ACTA2, BMP4*), and ectoderm (*BMP4* and *PAX6*) genes. Moreover, an in‐depth gene expression analysis for pluripotency was performed by PluriTest assay.^20^ hiPSCs characterization assessment is shown in Figure S1.

### 2.3 RNA extraction, RT‐PCR and qRT‐PCR

Total RNA was extracted using TRIzol reagent (Thermo Fisher Scientific) and 1 µg RNA was used for retro‐transcription using the High‐capacity cDNA Reverse Transcription Kit (Thermo Fisher Scientific). For SeV detection, half µg of cDNA was used for standard PCR reaction. However, qRT‐PCR was used for gene expression quantification using 1 µL of the RT reaction and the Power SYBR Green master Mix (Applied Biosystems). Gene expression levels were normalized to Glyceraldehyde 3‐phosphate dehydrogenase (*GAPDH*) housekeeping gene. qRT‐PCR was performed by StepOnePlus™ Real‐time PCR System (Applied Biosystems). A list of primers is provided in Table S1.

### 2.4 Immunohistological analysis and alkaline phosphatase staining

Cells were fixed with 4% (vol/vol) paraformaldehyde (PFA) and subjected to immunostaining using the following primary antibodies: NANOG (1:1000; rabbit polyclonal, Abcam), OCT4 (1:400 mouse monoclonal, STEMCELL Technologies) for pluripotency, and BRACHYURY (1:20 goat polyclonal, R&D systems), SOX17 (1:20 goat polyclonal, R&D systems) and NESTIN (1:1000 mouse monoclonal, STEMCELL Technologies) for the three germ layers detection. After incubation with primary antibodies, cells were incubated with Alexa‐Fluor‐647, ‐594 and ‐488 conjugated secondary antibodies (all from Thermo Fisher Scientific) for 1 hour at 37°C. Nuclei were counterstained using 1 µg/mL Hoechst 33528 (Thermo Fischer Scientific). Microscopy was performed using imaging systems (DMi8), filter cubes and software from Leica microsystems. AP staining was performed using the 1‐Step NBT/BCIP (Thermo Fisher Scientific). Fluorescence quantization was achieved by measurement of the corrected total cell fluorescence (CTCF = Integrated Density – [Area of selected cell × Mean fluorescence of background readings]). Five cells/condition were randomly selected for analysis.

### 2.5 Western blotting

For western blot analysis the cells were lysed in RIPA buffer (Sigma‐Aldrich) supplemented with Halt™ Protease Inhibitor Cocktail and Halt™ Phosphatase Inhibitor Cocktail (both from Thermo Fischer Scientific). The protein concentration was determined using a standard Bradford assay and separated by electrophoresis on acrylamide/bisacrylamide precast gels Mini‐PROTEAN TGX (Bio‐Rad), followed by transfer to the nitrocellulose membrane. Membranes were incubated with the following primary antibodies: anti: anti‐SQSTM1 (Abcam), anti‐RAB17, anti‐phospho HSPB1 (Ser82) and anti‐phospho SQSTM1 (Thr269/Ser272) (Cell Signalling), and next with horseradish peroxidase conjugated secondary antibody anti‐mouse IgG and anti‐rabbit IgG (Jackson ImmunoResearch). However, ɣ‐TUBULIN‐HRP conjugated (Santa Cruz, Biotechnology) or ACTIN (Santa Cruz Biotechnology) were used as internal loading control. Proteins were detected by Clarity™ Western ECL Blotting Substrates (Bio‐Rad).

### 2.6 Treatment of hESCs and hiPSC lines with retinoic acid and BMS493

For RAR pathway modulation, cells were treated for 24 hours either with 0.5 µM RA or with 5 µM BMS493 (a pan‐retinoic acid receptor inverse agonist) or with a combination of RA and BMS493, directly diluted in the culture media. For EBs formation assay, cells were dissociated into single cells using StemPro Accutase (Thermo Fisher Scientific) and cultured for 7 days on poly (2‐hydroxyethyl methacrylate) (Sigma‐Aldrich) – coated dishes in mTeSR1 medium supplemented with 10 µM of the Rho‐kinase inhibitor Y‐27632 (Selleckchem) for the first three days. At day 7, floating EBs were transferred on 5 µg/mL Biolaminin 521LN (Biolamina)‐coated plates and cultured in adhesion for additional thirteen days in medium consisting of DMEM/F12 containing 20% knockout serum replacement (KSR, Thermo Fisher Scientific), 1% Glutamax (Thermo Fisher Scientific), 1% non‐essential Amino Acids (Thermo Fisher Scientific), 100 µM 2‐mercaptoethanol and 0.5% penicillin and streptomycin.

### 2.7 Proteomic and phosphoproteomic analysis: Strategy overview

An integrated strategy that combines enzymatic digestion, isobaric mass tag labelling, a selective affinity technique which uses metal oxide affinity material (MOA) for phosphopeptides enrichment, peptides fractionation by strong cation exchange, and nanoLC coupled with high resolution tandem mass spectrometry was adopted. A total of 8 samples (200 µg each), four biological replicates per each cell line, hESCs and hiPSC‐1 respectively, were prepared for phosphoproteomics and proteomics analyses. The core of the strategy is represented by the use of isobaric tags, allowing for relative quantification and consequent identification of differentially expressed proteins (DEPs) between hESCs and hiPSC‐1 lysates. The total area under the chromatograms, the number of proteins and the number of sequenced peptides obtained from single injections were evaluated to estimate protein amounts per sample (Table S2): about 200 µg per sample was handled. These results were further used to ensure that the subsequent labelling reaction would be performed on a comparable amount of starting material. Moreover, nLC‐MS/MS analyses were also carried out after TMT labelling^21^ (Table S3): normalized median values per each TMT channel were evaluated. Thus, all the samples were combined by mixing different volumes into a single *master* sample which was further processed for phosphopeptides enrichment; in particular, 90% of the volume was used for the sample labelled 129N; 95% of the volume was used for the samples labelled 127C, 128N, 128C, 129C, 130N and 126, respectively; the whole volume was instead used for both the samples labelled 127N and 130C. Further purification and fractionation steps were needed before nLC‐MS/MS analysis for proteins relative quantification. Detailed Experimental Procedures for proteomics and phosphoproteomics‐enrichment sample preparation and mass spectrometric analysis of hiPSC‐1 and hESCs are provided in Supplementary material and methods section.

### 2.8 Microarray procedure

Total RNA was extracted using the Stratagene Absolutely RNA kit and resuspended in RNase‐free water. Spectrophotomeric determination of purified RNA yield was performed using the NanoDrop (Thermo Scientific), while total RNA quality was measured using the BioAnalyzer 2100 (Agilent Technologies). Antisense RNA (aRNA) was synthesized, amplified and purified using the Illumina TotalPrep RNA Amplification Kit (Ambion) following the manufacturer's instructions. For microarray, purified aRNA was hybridized to the Human HT‐12v4 Expression BeadChip Kit (Illumina). Samples were scanned on the iSCAN system (Illumina). The output file was statistically analysed.

### 2.9 Statistical analysis

For proteomic data, statistical analysis was carried out by using both GraphPad Prism (version 7.00 for Mac, GraphPad Software, La Jolla, California USA, www.graphpad.com) and Excel 2011 (version 14.0.0 for Mac, Microsoft; Redmond, WA) software. However, *t*‐tests were produced in Excel 2011 while Benjamini–Hochberg corrections for *q‐value* calculation were run through the Prism. Reporter ion intensities from the reference sample (*m/z* 126) were used as the denominator in both the phopshopeptide and the protein ratio calculations (two different ID lists). Both the protein and the phosphopeptide ratios from four biological replicates per class (N = 4 hESCs; N = 4 hiPSC‐1) were compared by student *t*‐test corrected for multiple hypothesis testing using the Benjamini‐Hochberg procedure (*q‐value* <0.05).^22^ Protein fold‐changes were determined by dividing protein's median fold‐changes (n = 4 replicates) of the two data sets. For microarray analysis, primary raw intensity data produced by Illumina iSCAN were imported in *R* statistical environment using *limma* package^23^ for background subtraction, quantile normalization and log_2_ transformation signal values. This procedure also removes the control probes, leaving only the regular ones. Moderated *t*‐test analysis with Benjamini and Hochberg (BH) multiple testing correction were used to identify differentially expressed genes (DEGs) between hESCs and hiPSC‐1. DEGs were selected by a fold‐change analysis of ≥1.5 and based on a *P* value cut‐off of ≤0.05. The identified DEGs were annotated in Gene Ontology (GO) and pathway analysis. Ingenuity Pathways Analysis (IPA; Ingenuity Systems, http://www.ingenuity.com website) was used for gene set enrichment and gene network analysis.

## 3 RESULTS

### 3.1 Whole proteome identification and classification of DEPs

**Figure 1:**
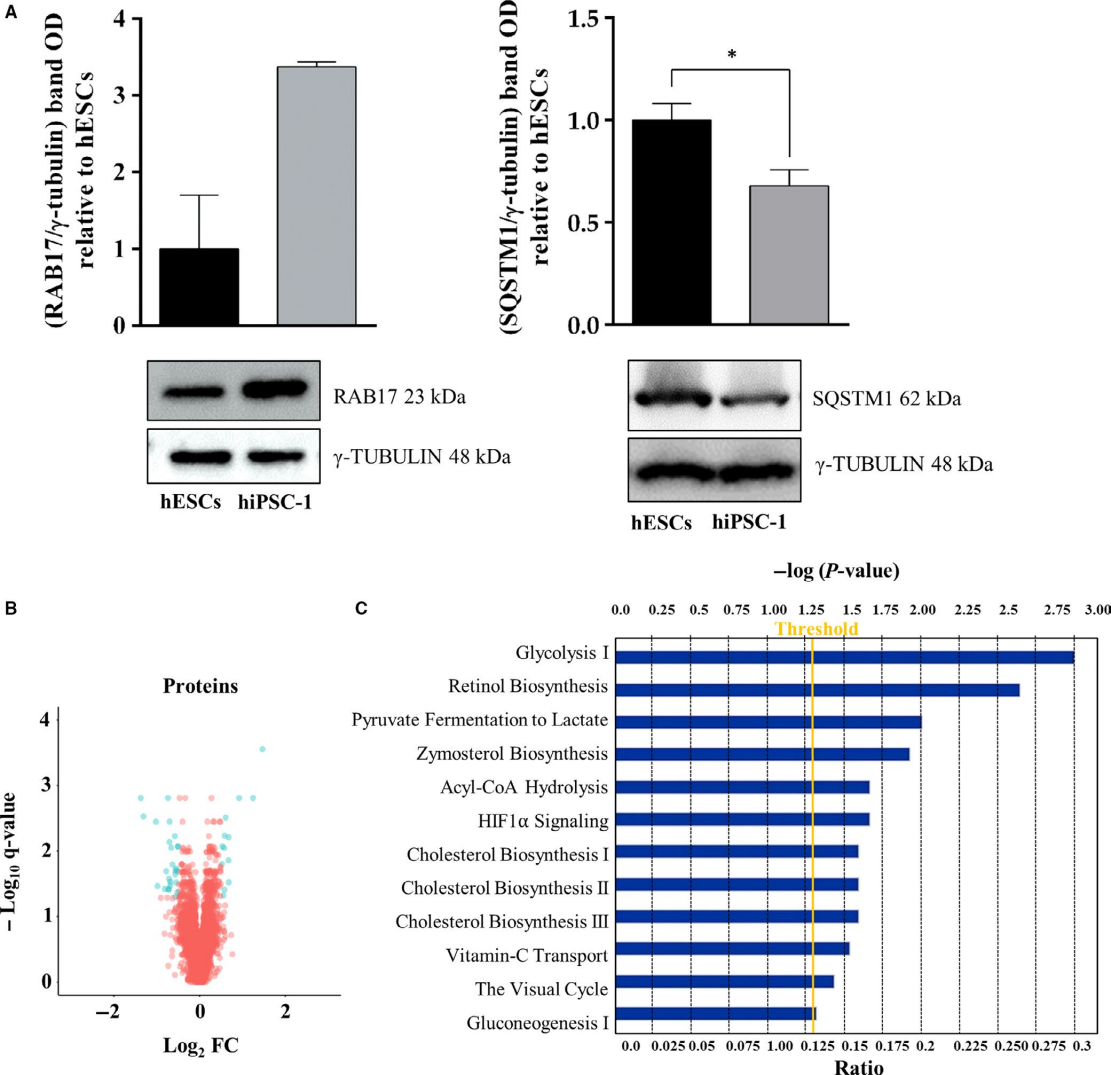
A, Western blot analysis for RAB17 (*P value* 0.3) and SQSTM1 (*P value* 0.03), up‐ and down‐regulated in hiPSC‐1 vs hESCs, respectively. Data are mean ± SEM from three independent biological replicates (*P < 0.05, t‐test). B, Volcano Plot of differentially dysregulated proteins (blue dots) between hiPSC‐1 vs hESCs. Unchanged proteins are represented as red dots. The x‐axis specifies the Log_2_ fold change (Fc) and the y‐axis specifies the *q*‐value negative logarithm in base 10. C, Ingenuity Pathway Analysis of specific signalling pathways enriched by the differentially expressed proteins. hESCs, human embryonic stem cells; hiPSCs, human induced pluripotent stem cells

Proteomics data resulting from nano‐Liquid Chromatography Tandem Mass Spectrometry (nLC‐MS/MS) allowed the identification and quantification of 3807 proteins between hiPSC‐1 and hESCs samples (Table S4). For proteomic and phosphoproteomic data, the same statistical cutoff was adapted and 230 statistically significant proteins (*q* < 0.05) were selected. Interestingly, by comparing hiPSCs vs hESCs, 13 proteins (CRYZ, CES1, SLC2A3, ALB, LBR, HM13, RAB17, SLC3A2, KRI1, RPRD1B, SLC25A1, SLC7A8, SLC6A6) were found enriched, according to a log_2_ fold change >0.5, and 25 proteins (PLIN2, IFITM2, PKM, GALNT3, KIAA1524, SQSTM1, STK26, PSMD5, ACOT9, ALDH16A1, CHCHD2, GMPR2, PGK1, SLC15A4, TSPAN3, LDHA, IFI30, P4HA2, TSPAN6, TCEAL4, HIST1H4A, HIST1H3A, DHRS4, MT1X, NLRP2) were down‐regulated according to the same log_2_ fold change (Table 1). Two proteins, RAB17 and SQSTM1, respectively up‐ and down‐regulated in hiPSC‐1 vs hESCs, were selected for biological validation via Western blot analysis (Figure 1A). Although the trend of expression is confirmed for both proteins, only the expression level of SQSTM1 resulted in statistically significant (*P value* 0.03). RAB17 is a member of the small GTPase superfamily and it has been linked to the down‐regulation of cell growth and proliferation.^24^ The protein resulted in up‐regulated hiPSCs vs hESCs in our proteogenomic comparison and, in a previous study, we demonstrated that hESCs have indeed a higher proliferation rate compared to hiPSCs as shown by cell cycle analysis.^11^ SQSTM1 is a hub molecule involved in several biological pathways, including autophagy that represented a highly conserved cellular process in ES cells supporting self‐renewal and regulating differentiation. Moreover, autophagy is activated during reprogramming of somatic cells to iPSCs.^25^

**Table 1:** Differentially expressed proteins in hiPSC‐1 vs hESCs

Accession	Gene	Description	hiPSC‐1/hESCs Fc	log_2_ Fc	q value
A6NP24	CRYZ	Quinone oxidoreductase (Fragment)	2.76	1.47	0.000
P23141	CES1	Liver carboxylesterase 1	2.37	1.25	0.002
P11169	SLC2A3	Solute carrier family 2, facilitated glucose transporter member 3	1.90	0.92	0.002
Q14739	LBR	Lamin‐B receptor	1.60	0.68	0.030
Q8TCT9	HM13	Minor histocompatibility antigen H13	1.60	0.68	0.006
Q9H0T7	RAB17	Ras‐related protein Rab‐17	1.60	0.67	0.014
F5GZS6	SLC3A2	4F2 cell‐surface antigen heavy chain	1.52	0.61	0.003
A0A0C4DGB6	ALB	Serum albumin	1.50	0.58	0.006
Q8N9T8	KRI1	Protein KRI1 homolog	1.50	0.58	0.039
Q9NQG5	RPRD1B	Regulation of nuclear pre‐mRNA domain‐containing protein 1B	1.49	0.57	0.009
P53007	SLC25A1	Tricarboxylate transport protein, mitochondrial	1.44	0.53	0.009
Q9UHI5	SLC7A8	Large neutral amino acids transporter small subunit 2	1.43	0.52	0.016
P31641	SLC6A6	Sodium‐ and chloride‐dependent taurine transporter	1.41	0.50	0.032
Q99541	PLIN2	Perilipin‐2	0.71	−0.50	0.009
H7BYV1	IFITM2	Interferon‐induced transmembrane protein 2 (Fragment)	0.70	−0.51	0.009
P14618	PKM	Pyruvate kinase	0.70	−0.52	0.043
E7EUL0	GALNT3	Polypeptide N‐acetylgalactosaminyltransferase 3	0.70	−0.52	0.020
Q8TCG1	KIAA1524	Protein CIP2A	0.69	−0.54	0.019
Q13501	SQSTM1	Sequestosome‐1	0.68	−0.55	0.030
Q9P289	STK26	Serine/threonine‐protein kinase 26	0.67	−0.57	0.020
Q16401	PSMD5	26S proteasome non‐ATPase regulatory subunit 5	0.67	−0.57	0.023
Q9Y305	ACOT9	Acyl‐coenzyme A thioesterase 9, mitochondrial	0.67	−0.58	0.006
Q8IZ83	ALDH16A1	Aldehyde dehydrogenase family 16 member A1	0.64	−0.64	0.016
Q9Y6H1	CHCHD2	Coiled‐coil‐helix‐coiled‐coil‐helix domain‐containing protein 2	0.63	−0.66	0.009
H0YMB3	GMPR2	GMP reductase	0.62	−0.69	0.030
P00558	PGK1	Phosphoglycerate kinase 1	0.62	−0.69	0.034
Q8N697	SLC15A4	Solute carrier family 15 member 4	0.61	−0.70	0.004
O60637	TSPAN3	Tetraspanin‐3	0.61	−0.71	0.007
P00338	LDHA	L‐lactate dehydrogenase A chain	0.61	−0.71	0.027
P13284	IFI30	Gamma‐interferon‐inducible lysosomal thiol reductase	0.61	−0.72	0.038
O15460	P4HA2	Prolyl 4‐hydroxylase subunit alpha‐2	0.60	−0.73	0.039
A0A087WZU5	TSPAN6	Tetraspanin‐6	0.60	−0.74	0.002
A2RQR6	TCEAL4	Transcription elongation factor A (SII)‐like 4 variant 1	0.58	−0.78	0.020
P62805	HIST1H4A	Histone H4	0.57	−0.81	0.038
P68431	HIST1H3A	Histone H3.1	0.51	−0.98	0.034
Q9BTZ2	DHRS4	Dehydrogenase/reductase SDR family member 4	0.49	−1.02	0.004
P80297	MT1X	Metallothionein‐1X	0.40	−1.31	0.003
A0A0G2JMG8	NLRP2	NACHT, LRR and PYD domains‐containing protein 2	0.39	−1.38	0.002

Note:

Among the selected 230 statistically significant proteins (q < 0.05), 13 (in dark grey) enriched in hiPSC‐1 vs hESCs according to a log_2_ fold change >0.5. 25 proteins (in light grey) were found down‐regulated in hiPSC‐1 vs hESCs according to the same log_2_ fold change.

hESCs, human embryonic stem cells; hiPSCs, human induced pluripotent stem cells.

Whole proteomic data analysis identified key regulators of pluripotency, including Sox15,^26^ up‐regulated in hiPSC‐1 vs hESCs, and OCT4, whose expression was similar in reprogrammed and ES cell lines (*q‐value* 0.43, fold‐change 0.91). Differentially regulated proteins are shown in a Volcano plot (neg log10, *q value*, vs fold change hiPSC‐1/hESCs) (Figure 1B). IPA of these proteins revealed the enrichment of the following signalling pathways: *Glycolysis I, Retinol Biosynthesis, Pyruvate Fermentation to Lactate, Zymosterol Biosynthesis, Acyl‐CoA Hydrolysis, HIF1α Signalling, Cholesterol Biosynthesis, Vitamin‐C Transport, The Visual Cycle, Gluconeogenesis I*, listed in Figure 1C. The superpathway of *Cholesterol Biosynthesis* resulted in enriched hiPSC‐1 vs hESCs, as demonstrated by the presence of two enzymes involved in the cholesterol biosynthesis, namely HMGCR (3‐Hydroxy‐3‐Methylglutaryl‐CoA Reductase) (Table S6) and LBR (Lamin B receptor) (Table S4), respectively down‐ and up‐regulated in hiPSCs.

### 3.2 Identification and classification of differentially expressed phosphoproteins

Phosphoproteome analysis allowed us to identify and quantify 5958 phophopeptides and 2623 phosphoproteins (Tables S5 and S6). Of these, 69 phophopeptides and 73 phosphoproteins were found statistically significant according to Student's *t*‐test with the Benjamini‐Hochberg correction (*q* < 0.05). 19 phosphopeptides and 14 phosphoproteins (SOX15, RPS8, DPF2, WBP4, SLC38A1, ADAR, CASK, PLEKHA6, BSG, SLC3A2, TRAM1, PLEKHG3, NCBP1, OVOL2) were found up‐regulated in hiPSC‐1 (based on log_2_ hiPSC‐1/hESCs >0.5). SOX15 was found over‐expressed within the hiPSCs‐1 digests according to a *q*‐value 0.02 and a fold‐change of 2.10. Conversely, 31 phosphopeptides and 20 phosphoproteins (PRKAR2A, BRAP, HMGCR, OSBPL8, CDC6, HSPB1, PPIL1, NPY1R, ANLN, THUMPD1, STX18, MGMT, ANP32B, CDC7, EFNB1, HTR1A, PDAP1, SQSTM1, SEMA4B, SPDL1) were found under‐expressed in hiPSC‐1 samples (based on log_2_ hiPSC‐1/hESCs <−0.5). Table 2 is relative to differentially expressed phosphoproteins.

**Table 2:** Differentially expressed phosphoproteins

Accession	Gene	Description	hiPSC‐1/hESCs Fc	log_2_ Fc	q value
O60248	SOX15	Protein SOX‐15	2.10	1.07	0.02
Q5JR95	RPS8	40S ribosomal protein S8	1.87	0.91	0.04
Q92785	DPF2	Zinc finger protein Ubi‐D4	1.85	0.89	0.04
O75554	WBP4	WW domain‐binding protein 4	1.62	0.70	0.04
F8VX12	SLC38A1	Sodium‐coupled neutral amino acid transporter 1 (Fragment)	1.58	0.66	0.03
P55265	ADAR	Double‐stranded RNA‐specific adenine deaminase	1.54	0.62	0.00
Q5JS72	CASK	Peripheral plasma membrane protein CASK	1.53	0.62	0.04
Q9Y2H5	PLEKHA6	Pleckstrin homology domain‐containing family A member 6	1.53	0.61	0.02
A0A087WUV8	BSG	Basigin	1.50	0.59	0.00
F5GZS6	SLC3A2	4F2 cell‐surface antigen heavy chain	1.49	0.58	0.04
G3XAN4	TRAM1	Translocating chain‐associated membrane protein 1	1.49	0.58	0.04
A1L390	PLEKHG3	Pleckstrin homology domain‐containing family G member 3	1.45	0.54	0.04
F2Z2T1	NCBP1	Nuclear cap‐binding protein subunit 1	1.44	0.52	0.04
Q9BRP0	OVOL2	Transcription factor Ovo‐like 2	1.41	0.50	0.03
P13861	PRKAR2A	cAMP‐dependent protein kinase type II‐alpha regulatory subunit	0.69	−0.53	0.03
J3KNN7	BRAP	BRCA1‐associated protein	0.69	−0.54	0.04
P04035	HMGCR	3‐hydroxy‐3‐methylglutaryl‐coenzyme A reductase	0.69	−0.54	0.04
Q9BZF1	OSBPL8	Oxysterol‐binding protein‐related protein 8	0.68	−0.55	0.04
J3KTI7	CDC6	Cell division control protein 6 homolog (Fragment)	0.67	−0.57	0.04
F8WE04	HSPB1	Heat shock protein beta‐1	0.67	−0.58	0.04
Q9Y3C6	PPIL1	Peptidyl‐prolyl cis‐trans isomerase‐like 1	0.65	−0.62	0.01
B4DKL9	NPY1R	Neuropeptide Y receptor type 1	0.64	−0.65	0.01
Q9NQW6	ANLN	Actin‐binding protein anillin	0.61	−0.70	0.04
Q9NXG2	THUMPD1	THUMP domain‐containing protein 1	0.61	−0.72	0.02
D6RC71	STX18	Syntaxin‐18 (Fragment)	0.60	−0.73	0.03
P16455	MGMT	Methylated‐DNA‐‐protein‐cysteine methyltransferase	0.60	−0.74	0.04
Q92688	ANP32B	Acidic leucine‐rich nuclear ph phoprotein 32 family member B	0.60	−0.74	0.03
O00311	CDC7	Cell division cycle 7‐related protein kinase	0.59	−0.77	0.04
P98172	EFNB1	Ephrin‐B1	0.56	−0.84	0.04
P08908	HTR1A	5‐hydroxytryptamine receptor 1A	0.53	−0.92	0.02
Q13442	PDAP1	28 kDa heat‐ and acid‐stable phosphoprotein	0.51	−0.98	0.02
E7EMC7	SQSTM1	Sequestosome‐1	0.51	−0.98	0.03
Q9NPR2	SEMA4B	Semaphorin‐4B	0.41	−1.29	0.00
Q96EA4	SPDL1	Protein Spindly	0.37	−1.45	0.02

Note:

In dark grey phosphoproteins found up‐regulated in hiPSCs‐1 vs hESCs (based on log_2_ hiPSC‐1/hESCs >0.5); in light grey phosphoproteins down‐regulated in hiPSC‐1 vs hESCs (based on log_2_ hiPSC‐1/hESCs <−0.5).

**Figure 2:**
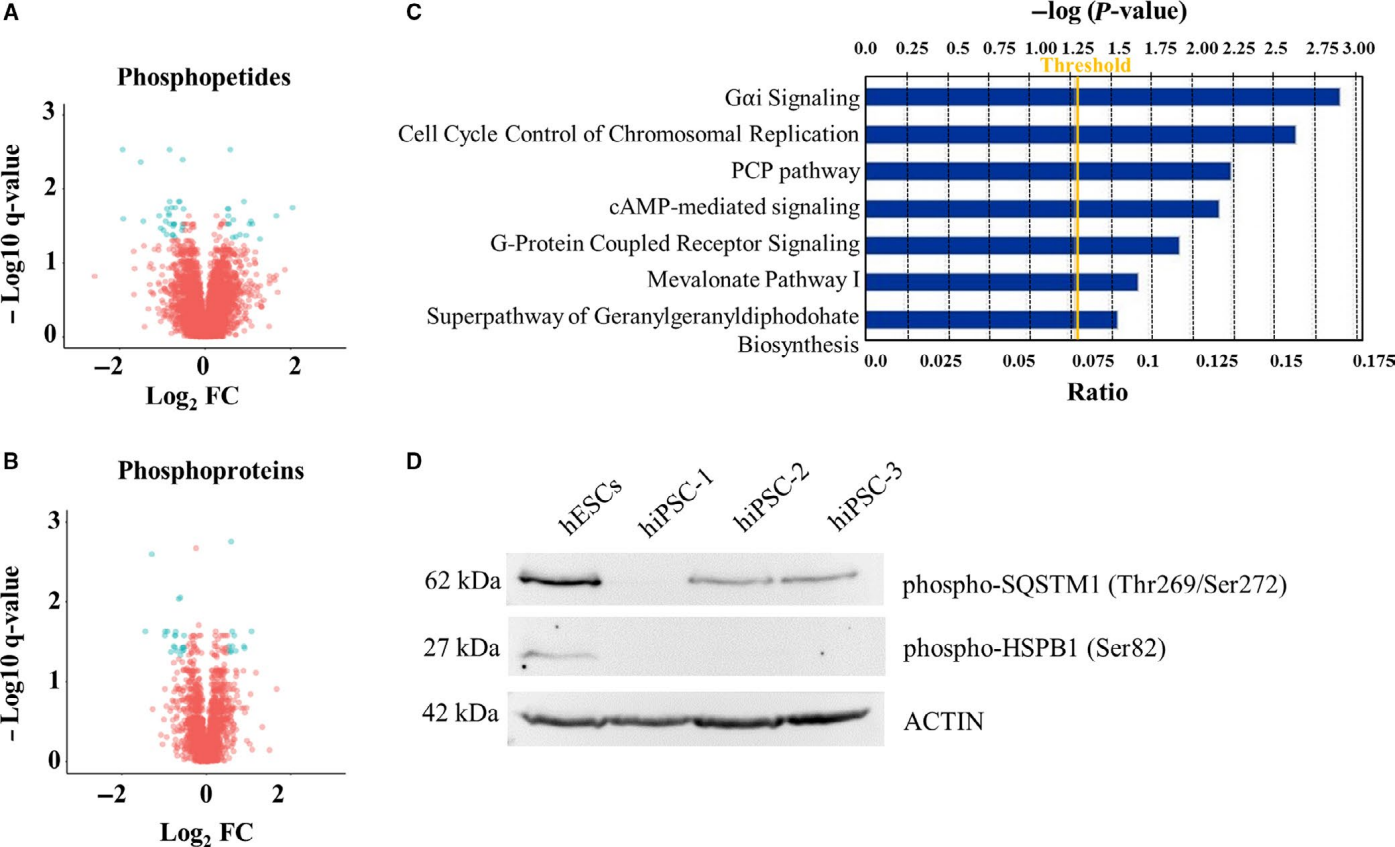
A, Volcano Plot representing differentially abundant phosphopetides between hiPSC‐1 and hESCs. B, Volcano Plot of differentially abundant phosphoproteins between hiPSC‐1 and hESCs. The *x*‐axis specifies the Log_2_ fold change (Fc) and the *y*‐axis specifies the *q*‐value negative logarithm in base 10. Differentially expressed phosphopeptides and phoshoproteins are represented as blue dots, while the unchanged ones are shown as red dots. C, Ingenuity Pathway Analysis showing signalling pathways enriched by the differentially expressed phosphoproteins. D, Western blot analysis of phospho‐HSPB1 (Ser82) and phospho‐SQSTM1 (Thr269/Ser272), both down‐regulated in hiPSCs (‐1, ‐2, and ‐3) vs hESCs. hESCs, human embryonic stem cells; hiPSCs, human induced pluripotent stem cells

Differentially abundant phosphopetides and phosphoproteins, plotted in Figure 2A and 2, account for the enrichment of *Gαi Signalling, Cell Cycle control of chromosomal replication, PCP Pathway, cAMP‐mediated Signalling and G‐Protein‐Coupled receptor Signalling* as shown by IPA analysis in Figure 2C. The expression of two phosphoproteins, the phospho‐HSPB1 (Ser82) and the phospho‐SQSTM1 (Thr269/Ser272), both down‐regulated in hiPSC‐1 vs hESCs, was validated via Western Blot analysis in hESCs and three independent hiPSCs lines (hiPSC‐1, hiPSCs‐2, and hiPSCs‐3), confirming the trend of expression obtained with the phoshoproteomic analysis (Figure 2D).

### 3.3 Transcriptome analysis for the identification and classification of DEGs

**Figure 3:**
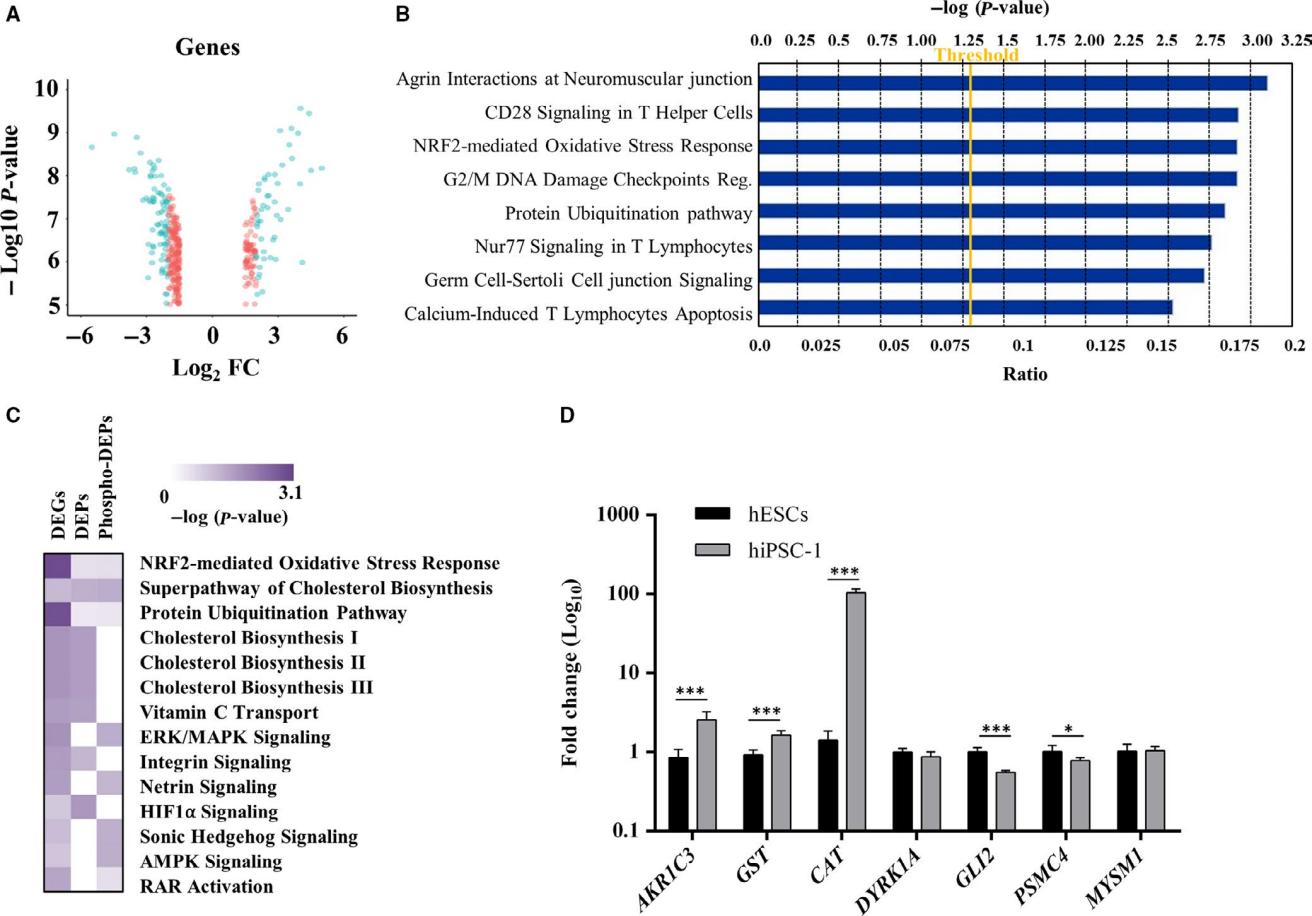
A, Volcano plot representing differentially expressed genes (blue dots) between hiPSC‐1 and hESCs; the unchanged genes are shown as red dots. The *x*‐axis specifies the Log_2_ fold change (Fc) and the *y*‐axis specifies the *q*‐value negative logarithm in base 10. B, Top canonical signalling pathways uncovered by Ingenuity Pathway Analysis. C, Heat Map representing color‐coded networks derived from the integrative transcriptomic, whole proteomic and phosphoproteomic analysis of hiPSC‐1 and hESCs (the colour intensity scale is proportional to the statistical significance of a specific pathway enriched in each dataset, – log (*P*‐value). D, Quantitative real‐time polymerase chain reaction analysis of the NRF2‐mediated Oxidative Stress Response (*AKR1C3*, *GST*, *CAT*), Sonic Hedgehog (*DYRK1A* and *GLI2*) and Protein Ubiquitination pathways (*PSMC4* and *MYSM1*) ‐ associated genes. Expression values are normalized to *GAPDH* and relative to hESCs. Data are mean ± SD from three independent experiments (*P < 0.05, ***P < 0.001, t‐test). hESCs, human embryonic stem cells; hiPSCs, human induced pluripotent stem cells

Comparative transcriptome analysis of hiPSC‐1 vs hESCs, followed by a functional annotation analysis, highlighted 433 DEGs (Figure 3A), 136 of which were up‐regulated and 297 down‐regulated in hiPSC‐1 vs hESCs (Table S7).

IPA analysis uncovered the top canonical pathways enriched in hiPSC‐1 such as *Agrin Interactions at Neuromuscular junction, CD28 Signalling in T Helper cells, NRF2‐mediated Oxidative stress response, G2/M DNA Damage checkpoint regulation, Protein Ubiquitination pathway, Nur77 Signalling in T Lymphocytes, Germ Cell‐Sertoli Cell junction Signalling, and Calcium‐Induced T lymphocytes Apoptosis* (Figure 3B).

### 3.4 Integrative whole proteome, phosphoproteome and transcriptome analysis

**Figure 4:**
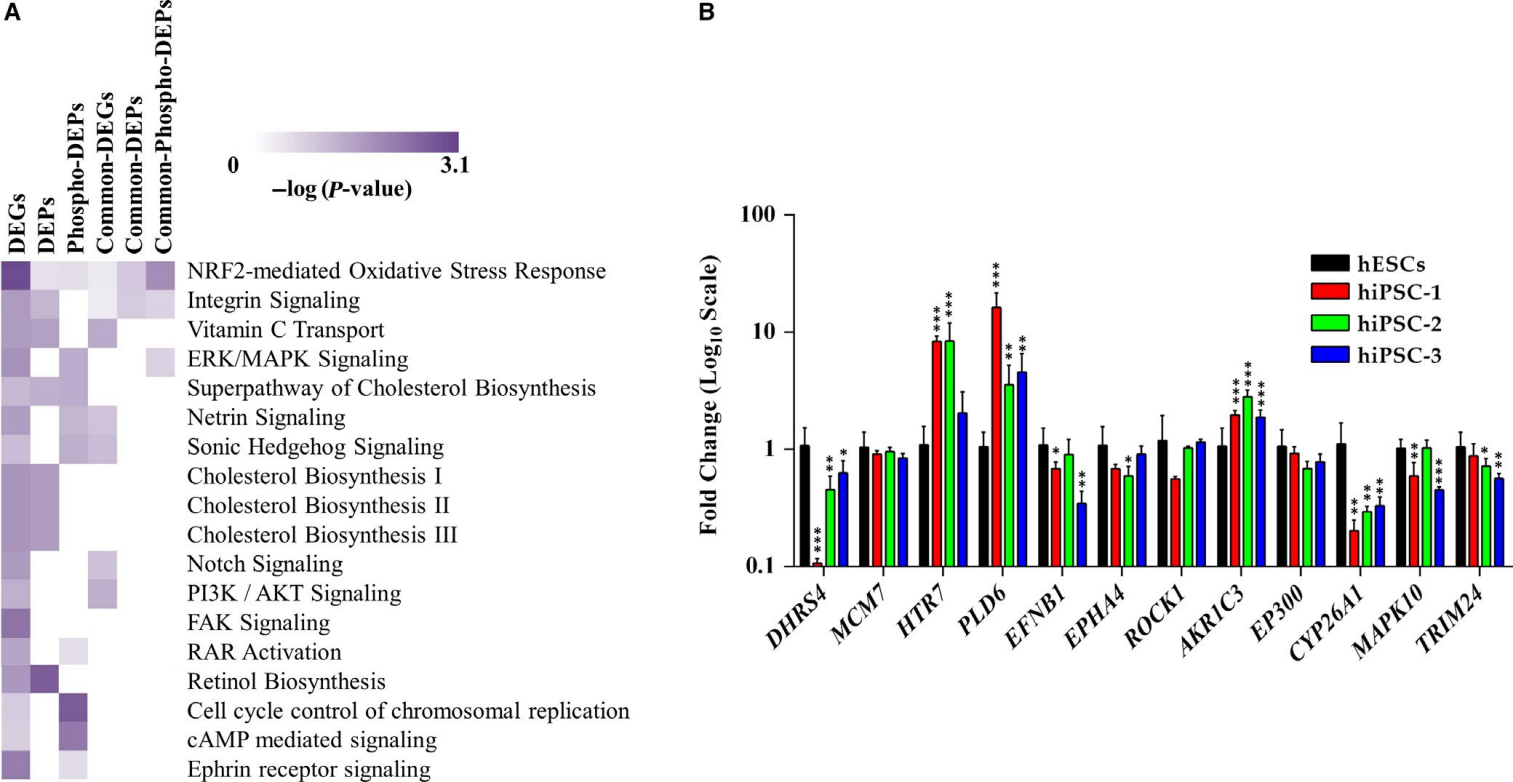
A, Heat Map representing signalling pathways exclusively and commonly enriched in our dataset and in the one coming from Phanstiel et al. B, Quantitative real‐time polymerase chain reaction analysis of some target genes associated with the exclusively enriched pathways. Expression values are normalized to *GAPDH* and relative to human embryonic stem cells. Data are mean ± SD from three independent experiments (*P < 0.05, **P < 0.01, ***P < 0.001, t‐test)

For a comprehensive proteogenomic analysis, we performed a ‘cross‐omics’ study using datasets encompassing mRNA, protein and phosphoprotein expression profiles obtained from hESCs and hiPSC‐1. Among the networks commonly shared, we identified the *NRF2‐mediated Oxidative Stress Response, Superpathway of Cholesterol Biosynthesis, and Protein Ubiquitination Pathway.* The complete list of signalling pathways identified is provided in Table S8. Interestingly, IPA comparison also uncovered a number of pathways enriched only in two out of three datasets given as input. In particular: (a) *Cholesterol biosynthesis (I, II, III), Vitamin C Transport, Integrin Signalling and HIF1α Signalling* are enriched in transcriptome and whole proteome datasets; (b) *ERK/MAPK Signalling, Netrin Signalling, Sonic Hedgehog Signalling, AMPK Signalling and RAR activation* are common among transcriptome and phosphoproteome datasets (Figure 3C). For biological validation of the integrative comparison the following genes were analysed by qRT‐PCR: *AKR1C3*, *GST*, *CAT* (NRF2‐associated), *PSMC4* and *MYSM1* (Protein Ubiquitination Pathway‐associated) (both pathways commonly enriched in all three datasets), *DYRK1A* and *GLI2* (Sonic Hedgehog Signalling‐associated) (shared by transcriptome and phosphoproteome datasets). The qRT‐PCR analysis confirmed the trend of expression of the proteogenomic data: *AKR1C3* (Fc 2.56), *GST* (Fc 1.64), *CAT* (Fc 104.69) significantly up‐regulated in hiPSC‐1 vs hESCs, *GLI2* (Fc 0.55) and *PSMC4* (Fc 0.79) significantly down‐regulated in hiPSC‐1 vs hESCs, the expression of *DYRK1A*, although down‐regulated in hiPSC‐1 (Fc 0.87), is not statistically significant. Finally, the expression level of *MYSM1* remains unchanged between the two lines (Figure 3D). Further, we used the cutoff applied in the present manuscript to analyse data from a similar work by Phanstiel et al^18^ Several pathways, such as those involved in the regulation of cell proliferation such as PI3K/Akt signalling, Sonic Hedgehog, Notch signalling, and in the maintenance of embryonic stem cell self‐renewal and pluripotency such as NRF, and ERK/MAPK signalling resulted commonly enriched (Figure 4A). Although the majority of pathways identified by this cross‐analysis are commonly shared by the two studies, some resulted exclusively enriched in our dataset, such as *Retinol Biosynthesis, Cell Cycle Control of Chromosomal Replication, cAMP‐mediated Signalling, and Ephrin Receptor Signalling, FAK Signalling, and RAR Activation pathway*. Further, we selected some target genes of these “exclusive” pathways (*DHRS4* as target of the *Retinol Biosynthesis pathway*, *MCM7* as target of the *Cell Cycle Control of Chromosomal Replication*, *HTR7* and *PLD6* for the *cAMP‐mediated signalling, EFNB1*, *EPHA4* and *ROCK1* for the *Ephrin Receptor signalling* and *AKR1C3*, *EP300*, *CYP26A1*, *MAPK10*, and *TRIM24* for *RAR Activation pathway*) and validated them by qRT‐PCR analysis in hESCs, and hiPSC‐1, hiPSC‐2, and hiPSC‐3 lines. *DHRS4* (average Fc 0.40), *EFNB1* (Fc 0.64), *EPHA4* (average Fc 0.73), *CYP26A1* (average Fc 0.28), *MAPK10* (average Fc 0.70) and *TRIM24* (average Fc 0.72) resulted down‐regulated in hiPSC vs ESCs. *MCM7* (Average Fc 0.9), *ROCK1* (Average Fc 0.91) and *EP300* (Average Fc 0.80) were not significantly dysregulated, *HTR7* (average FC 6.26), *PLD6* (average FC 8.14), and *AKR1C3* (average FC 2.22) resulted up‐regulated in hiPSCs vs hESCs (Figure 4B). A complete list of Signalling pathways identified is provided in Table S9.

### 3.5 Role of the RAR activation pathway and its modulation on the phenotype of human ESCs and human PSCs

**Figure 5:**
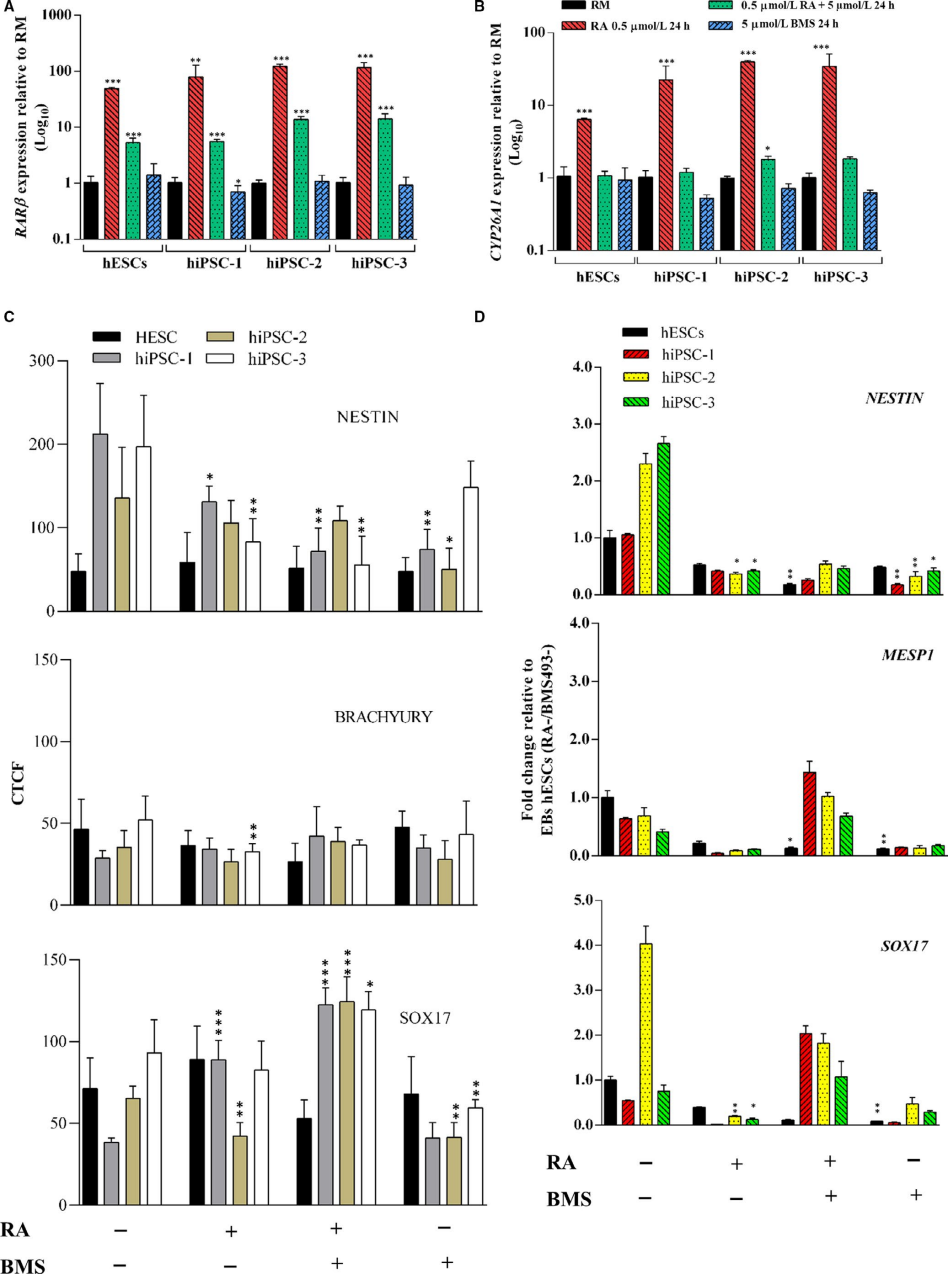
A, Quantitative real‐time polymerase chain reaction (qRT‐PCR) analysis of RARβ. B, qRT‐PCR analysis of CYP26A1 in human embryonic stem cells (hESCs) and human induced pluripotent stem cells (hiPSCs) (hiPSC‐1, ‐2, and ‐3) treated with RA (RA+) or with BMS493 (BMS493+) or with both (RA+/BMS493+); expression values are normalized to *GAPDH* and relative to regular medium (RM) (mTeSR1 medium). C, Corrected total cell fluorescence of markers specific of each germ layer: Nestin (ectoderm), Brachyury (mesoderm) and Sox17 (endoderm) in EBs derived from hESCs and hiPSCs cell lines (‐1, ‐2, and ‐3) cultured in RM and in medium supplemented for 24 h with 0.5 µM RA, 5 µM BMS493 or with both molecules. Bars represents measurements derived from 5 cells for each condition (mean ± SD). D, qRT‐PCR analysis of *NESTIN*, *MESP1* (mesoderm marker) and *SOX17* in EBs derived from hESCs and hiPSC‐1, ‐2, and ‐3 cultured in RM and in medium supplemented for 24 h with 0.5 µM RA, 5 µM BMS493 or with both molecules. Expression values are normalized to *GAPDH* and relative to EBs derived from untreated hESCs. Data are showed as mean ± SD from three independent experiments (*P < 0.05, **P < 0.01, ***P < 0.001, t‐test)

Among the pathways identified as differentially regulated in human ESCs and iPSC‐1, we selected the *RAR Activation signalling*, as retinoids including Vitamin A and its derivatives have been widely associated with embryonic development and differentiation.^27^, ^28^ Here, we evaluated the effects on hESCs and hiPSC‐1, ‐2, and ‐3 of two molecules, the all‐trans Retinoic Acid (RA) which functions as positive regulator of the RAR pathway, and BMS493, a powerful Pan‐retinoic acid receptor (pan‐RAR) antagonist that enhances nuclear corepressor (NCoR) interaction with RARs.^29^ Its binding induces analogous conformational changes in all RAR types (RARα, RARβ and RARγ) inactivating the transcription of target genes. For RAR pathway modulation, cells were treated either with RA or with BMS493 or with a combination of RA and BMS493 together for 24 hours. When exposed to RA, the expression of a direct target of Retinoic Acid, *RARβ*, increased in all cell lines while the combination of RA and BMS493 induced a reduction in its expression but still higher compared to untreated cells (Figure 5A). The exposure to RA triggered a 6‐fold increased expression of *CYP26A1*, a target of RA in hESCs and a 22‐ to 40‐fold increased expression in hiPSCs (Figure 5B). Endogenous production of RA is unlikely to take place in ES cell,^30^ although these cells express RARx, RXRs and Crabp1 required for RA response and for regulation of the expression of its targets such as RARβ and CYP26A1 in a time and dose‐dependent manner. This might explain why the treatment of hESCs and hiPSCs‐2 and ‐3 with BMS493 alone does not affect the expression of RARβ and CYP26A1 compared to cells cultured in Regular Medium. Exposure to either RA or BMS493 or to a combination of both molecules did not induce a significant variation in any of the other genes accounting for the enrichment of the RAR pathway such as *AKR1C3, MAPK10, TRIM24* with the exception of *EP300* resulted in induced hESCs treated with BMS493 for 24 hours (Figure S2). Further we analysed the effect of RAR modulation on differentiation potential of hESCs and hiPSCs lines. After treatment with RA or BMS493 or both, cells were induced to differentiation by Embryoid Body (EB) formation assay. Even though the overall capability of hESCs and hiPSCs (‐1, ‐2, and 3) to differentiate were maintained, the expression levels of specific germ layer‐associated genes such as *NESTIN* (ectodermal differentiation), *BRACHYURY* (mesodermal differentiation), and *SOX17* (endodermal differentiation) were differentially affected in response to RA, BMS or both used in combination. While the differentiation ability of hESCs was not significantly affected by the treatment with RA or BMS493 or both in combination as shown by immunofluorescence quantification, the hiPSCs lines (‐1, ‐2, and ‐3) showed an enhanced expression of *SOX17* when they were simultaneously exposed to RA and BMS493 (RA+/BMS493+) as shown by immunofluorescence (Figure S3) and its quantification (Figure 5C). qRT‐PCR analysis for *NESTIN*, *MESP1* and *SOX17* expression showed a reduction of these markers in hESCs in all the three conditions tested (RA+; BMS493+; RA+/BMS493+); conversely, although the hiPSC lines showed a similar trend when exposed either to RA or BMS493, they completely recovered their endodermal and mesodermal differentiation potential when simultaneously treated with both molecules (RA+/BMS493+), while the ectodermal differentiation capability did not significantly changed (Figure 5D).

## 4 DISCUSSION

Recent breakthroughs in stem cells research allowed to envision a scenario in which a stem cell‐based therapy might become reality. Particularly, the findings that differentiated somatic cells can be reprogrammed back to a pluripotent state and subsequently differentiated towards several cell types showed the feasibility of using the hiPSCs in a clinical setting. Although hiPSCs share a number of characteristics with hESCs, many fundamental questions around their molecular and functional equivalence still remain unanswered. Here we used a combined multi – “omics” strategy for proteogenomic analysis of hESCs and hiPSC, highlighting differences between these cells. IPA analysis of DEPs and transcripts demonstrated the enrichment of signalling pathways mostly associated to pluripotency and cellular metabolism, whose role goes beyond energy production being crucial in cell fate regulation.^31^ The *HIF1α signalling*, for instance, enriched in hiPSC‐1 vs hESCs, mediates the effects of oxygen concentration on stem cells proliferation and differentiation by induction of the hypoxia‐associated genes.^32^ Accordingly, in response to oxygen fluctuations, oxygen signalling controls the balance between pluripotency and differentiation. Highly pluripotent ESCs associate with hypoxia, high levels of glucose consumption and large amount of lactate production.^33^, ^34^ The *NRF2‐mediated oxidative stress response pathway,* commonly enriched in hESCs and hiPSC‐1, has been described as a key regulator of self‐renewal ability of hESCs playing a role in re‐establishment of pluripotency during cellular reprogramming.^35^, ^36^ Although PSCs rely more on glycolysis for energy and rapid cell proliferation, the tricarboxylic acid cycle in PSCs provides intermediate metabolites such a citrate and α‐ketoglutarate that are siphoned for lipids and amino acid biosynthesis.^37^ We found that LDHA and two glycolytic enzymes, phosphoglycerate kinase 1 (PGK1) and the pyruvate kinase (PKM) are down regulated in hiPSC‐1 compared to hESCs, while the mitochondrial carrier SLC25A1, encoding for a trycarboxylate protein that functions as transporter of the mitochondrial citrate into the cytosol, results up‐regulated in hiPSC‐1. Moreover, there are evidences suggesting a co‐existence of both oxidative and glycolytic metabolisms in stem cells.^38^ Cholesterol contributes to the maintenance of self‐renewal in ESCs by regulation of LIF‐induced signalling, and its derivatives, such as glucocorticoids, regulate cell fate during early development and seem to control cardiac differentiation in mESCs.^39^ Panopoulus et al^40^ demonstrated that the level of some unsaturated fatty acids such as arachidonic acid, implicated in the cholesterol homeostasis regulation, differs quantitatively between iPSCs and ESCs.^41^ Another pathway that resulted enriched in hiPSC‐1 vs hESCs is represented by the anti‐oxidant system as shown by the over‐expression in hiPSC‐1 of the glucose transporter SLC2A3, previously associated with an anti‐oxidant role.^42^

To further strengthen our findings, we used the cutoff applied in this study to analyse data from Phanstiel et al^18^ Several signalling pathways were commonly enriched in both studies, while others (*Retinol Biosynthesis, Cell cycle Control of Chromosomal Replication, cAMP‐mediated Signalling, Ephrin Receptor Signalling, FAK Signalling, and RAR Activation Signalling*) resulted exclusively enriched in our datasets and validated by qRT‐PCR analysis. Among these exclusive pathways, we focused on the *RAR Activation Pathway* since Retinoic acid and its receptors are classically linked both to embryonic development^43^, ^44^ and to pluripotency maintenance.^28^, ^45^ Subtle regulation of RA signalling is fundamental for the PSCs in order to choose between self‐renewal and differentiation programmes, as demonstrated by the fact that hESCs and hiPSCs behave differently when they undergo to treatment with Retinoic acid or BMS493. To investigate the role of RAR pathway, we first looked at the expression level of its target genes such as *AKR1C3*, *MAPK10*, *EP300* and *TRIM24* in hESCs and hiPSC‐1, ‐2, and ‐3 cultured in regular medium (mTeSR1), showing an increased expression of *AKR1C3* in hiPSCs lines compared to hESCs, while the expression levels of *MAPK10*, *EP300*, *CYP26A1* and *TRIM24* had an opposite trend. When cells were treated with RA the expression of *CYP26A1* was dramatically increased in hiPSC lines compared to hESCs. These data suggest that hiPSCs are more sensitive to RA than hESCs. This greater sensitivity of hiPSCs to RA can explain the higher expression of *CYP26A1*, a member of the cytochrome P450 family that controls the levels of RA by its oxidation to a less biologically active form, aiming to keep the pathway in a balanced activity. This hypothesis is supported by previously published data demonstrating that *CYP26A1* ‐/‐ ESCs exhibit an increase of intracellular level of RA accompanied by a reduction of the differentiation capability respect to wild‐type ESCs.^46^ hESCs and hiPSCs showed a different propensity to enter a specific lineage commitment in response to treatment (RA+ or BMS493+ or RA+/BMS493+). hiPSC lines treated with RA or BMS493 alone show a slight resistance to the endodermal lineage. A complete re‐establishment of hiPSCs differentiation potential is instead observed when cells are simultaneously treated with RA and BMS493, supporting the fact that a synergistic action of both molecules is sufficient to restore the activity of the pathway.

In conclusion, our data indicate that the molecular correspondence between hiPSCs and hESCs is not exactly linear, as demonstrated by the presence of specific pathways representative for each cell line. The discovery of pathways enriched in hiPSC‐1 vs hESCs and the identification of pathways exclusively present in our dataset are in agreement with the view that pluripotency is an extremely complex and multifaceted phenomenon, with peculiarities that are characteristic of each pluripotent cell type, and that hESCs and hiPSCs cannot be considered equivalent from a functional point of view. Our data provide evidence that reprogrammed cells possess a unique molecular signature that can have functional and phenotypic consequences when a given pathway is modulated. Future investigations of the identified pathways and their relative components will provide new insights into the complex mechanisms of pluripotency and self‐renewal of reprogrammed cells and will give the opportunity to understand how molecular variations can impact the phenotypic and functional behaviors of reprogrammed stem cells.

## CONFLICTS OF INTEREST

The authors declare no conflict of interest.

## AUTHOR CONTRIBUTIONS

GC conceived the study; EIP, SS, DT, MTD, GS, MG and GS performed the experiments; analysed the data; EIP, SS, DT, GS analysed the data and performed interpretation; GS and DT conducted the bioinformatic and statistical analysis; EIP, SS, DT, GC and GS wrote this manuscript; GC and GS reviewed and edited the final version of this article; GC provided the funds and materials. All authors have read and approved the final edition of this manuscript.

## ETHICAL APPROVAL

The use and inclusion of human samples was approved by the Ethics Committee of the ‘Magna Graecia’ University of Catanzaro and the Azienda Ospedaliero – Universitaria “Mater Domini”. Skin biopsy and whole blood withdrawal were obtained from three individuals without clinical evidences for any specific disease after written informed consent from the donor.
