# Supplementary figures and images for: Comprehensive proteogenomic analysis of human embryonic and induced pluripotent stem cells
Source: J Cell Mol Med. 2019 Jun 25;23(8):5440–53. doi: 10.1111/jcmm.14426 (PMC6653499; doi:10.1111/jcmm.14426)

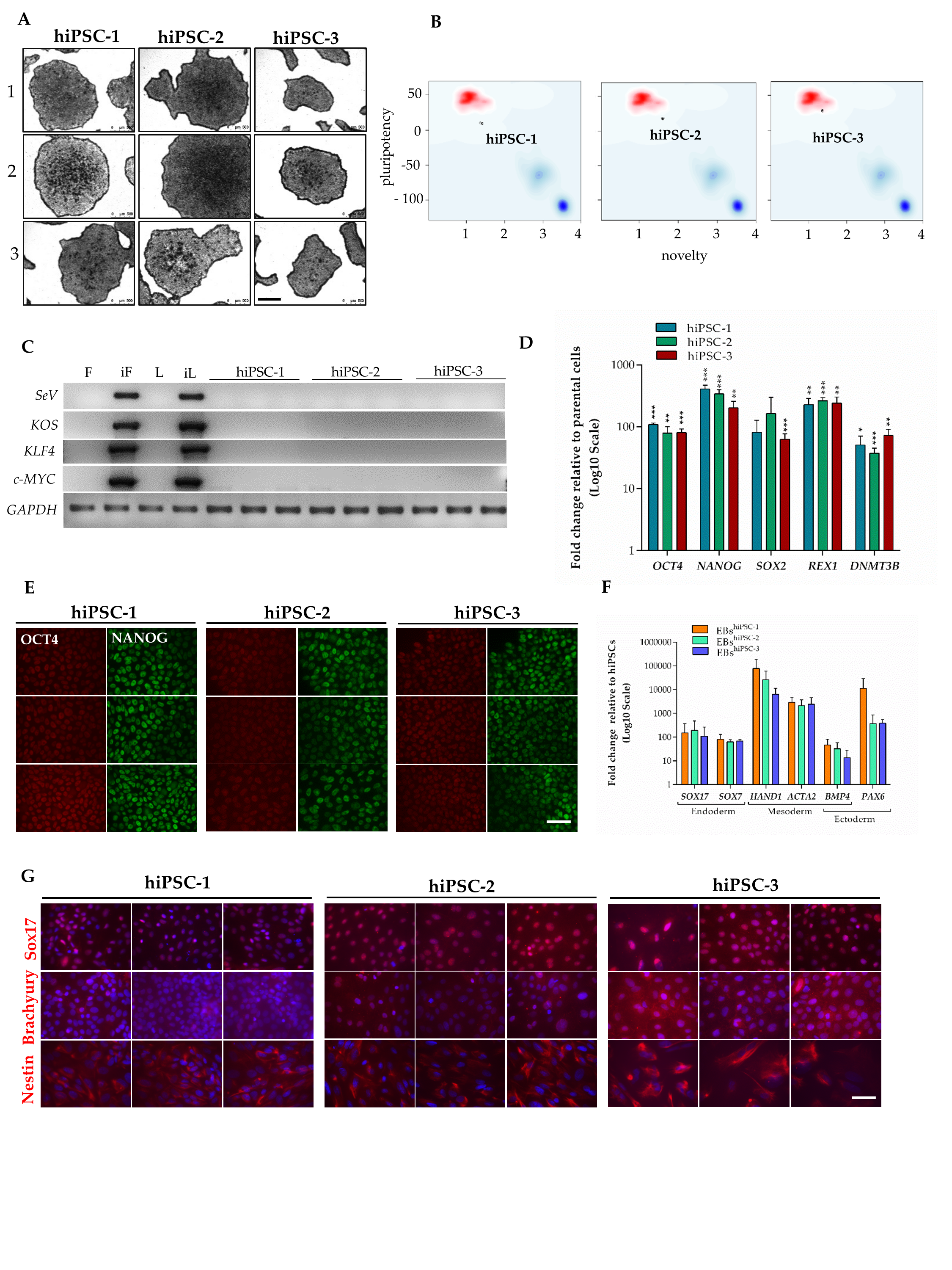

Supplement: Supplementary file 1 [file JCMM-23-5440-s001.tif]

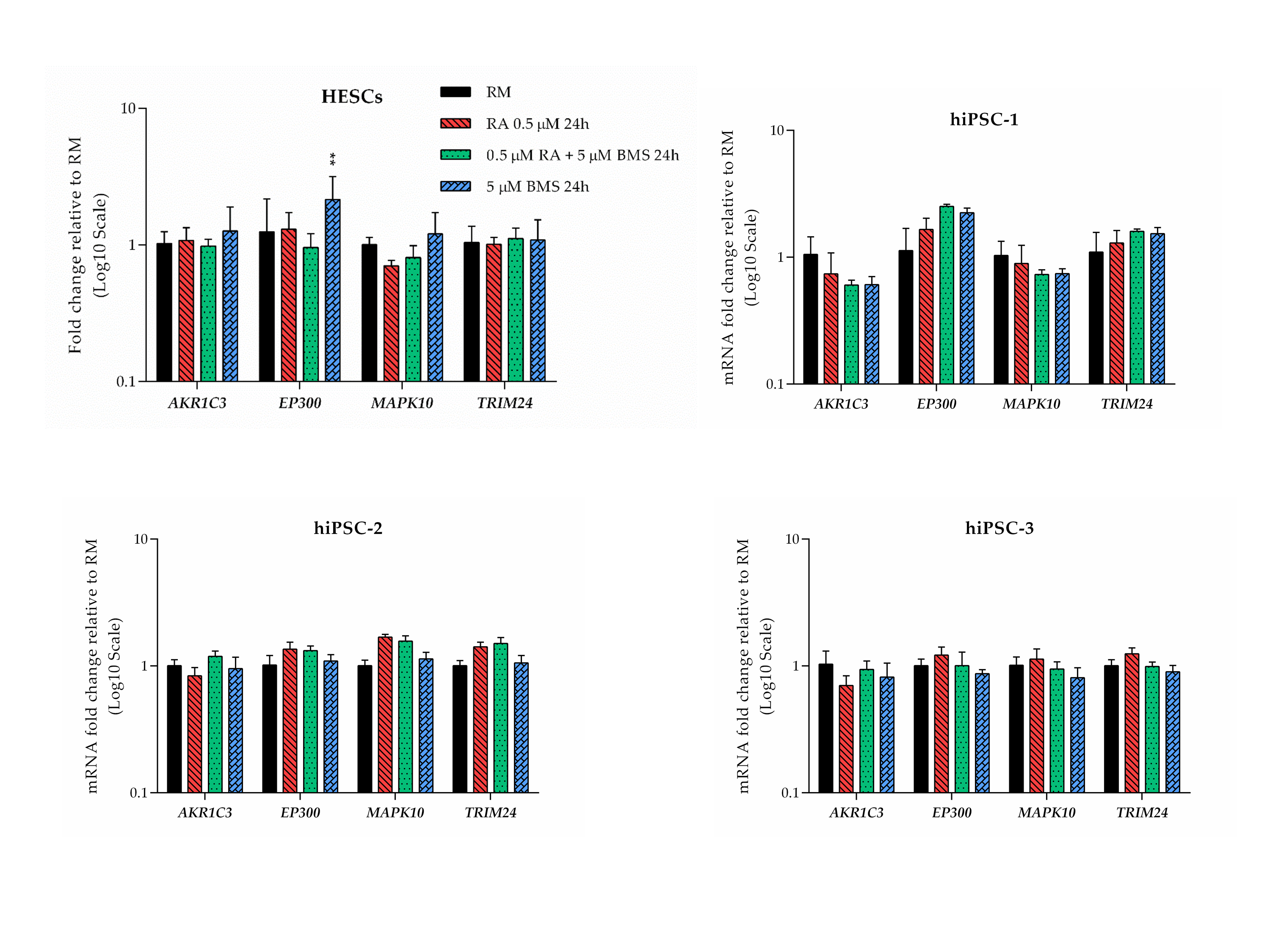

Supplement: Supplementary file 2 [file JCMM-23-5440-s002.tif]

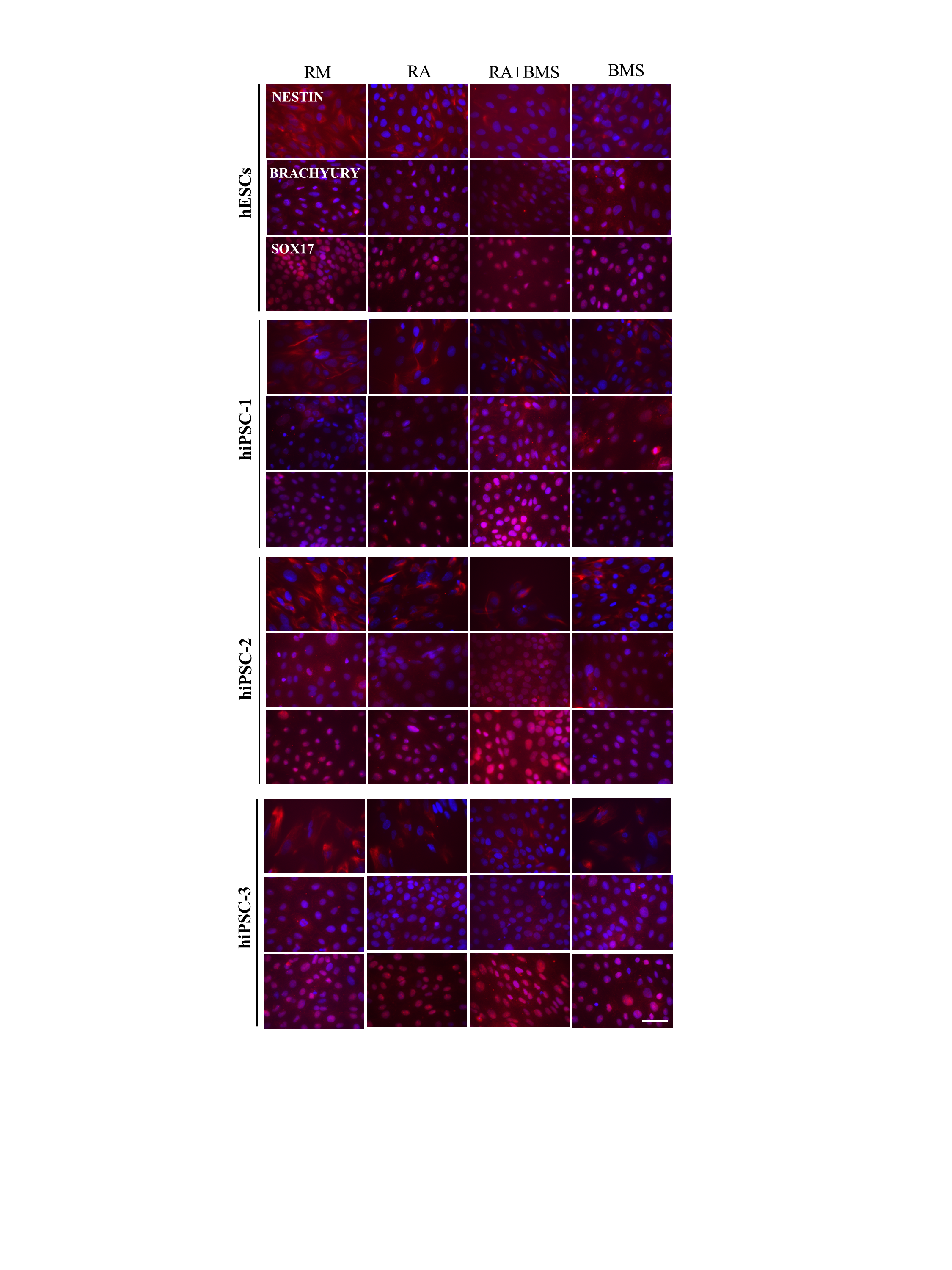

Supplement: Supplementary file 3 [file JCMM-23-5440-s003.tif]
