# Supplementary material for: Comprehensive proteogenomic analysis of human embryonic and induced pluripotent stem cells
Source: J Cell Mol Med. 2019 Jun 25;23(8):5440–53. doi: 10.1111/jcmm.14426 (PMC6653499; doi:10.1111/jcmm.14426)
Supplement: Supplementary file 4 [file JCMM-23-5440-s004.docx]

**Primers used for PCR and qRT-PCR.**

| **Gene** | **Primer forward** | **Primer reverse** |
| --- | --- | --- |
| *GAPDH* | TCCTCTGACTTCAACAGCGA | GGGTCTTACTCCTTGGAGGC |
| *OCT4* | GACAGGGGGAGGGGAGGAGCTAGG | CTTCCCTCCAACCAGTTGCCCCAAAC |
| *NANOG* | TGCAAGAACTCTCCAACATCCT | ATTGCTATTCTTCGGCCAGTT |
| *SOX2* | GGGAAATGGGAGGGGTGCAAAAGAGG | TTGCGTGAGTGTGGATGGGATTGGTG |
| *REX1* | ACCAGCACACTAGGCAAACC | TTCTGTTCACACAGGCTCCA |
| *DNMT3B* | CCATGAAGGTTGGCGACAA | TGGCATCAATCATCACTGGATT |
| *SeV** | GGATCACTAGGTGATATCGAGC | ACCAGACAAGAGTTTAAGAGATATGTATC |
| *KOS-tg** | ATGCACCGCTACGACGTGAGCGC | ACCTTGACAATCCTGATGTGG |
| *Klf4-tg** | TTCCTGCATGCCAGAGGAGCCC | AATGTATCGAAGGTGCTCAA |
| *cMYC-tg** | TAACTGACTAGCAGGCTTGTCG | TCCACATACAGTCCTGGATGATGATG |
| *SOX17* | CGCACGGAATTTGAACAGTA | GGATCAGGGACCTGTCACAC |
| *SOX7* | TGAACGCCTTCATGGTTTG | AGCGCCTTCCACGACTTT |
| *HAND1* | CCAGCTACATCGCCTACCTG | CCGGTGCGTCCTTTAATCCT |
| *ACTA2* | GTGATCACCATCGGAAATGAA | TCATGATGCTGTTGTAGGTGGT |
| *BMP4* | CCTGTTGTGTGCCCACTGAAC | ATCTCAGCGGCACCCACAT |
| *PAX6* | CAGCTTCACCATGGCAAATAA | ATCATAACTCCGCCCATTCA |
| *AKR1C3* | TACAATGAGCAGCGCATCAG | CTGGTAGACATCAGGCAAAGC |
| *GSTO2* | CTATTCTCACAGGACCCGCC | GATCAGTTGACATTGGCTGGT |
| *CAT* | CTGACTATGGCATCCGGGAT | CTGGGATGAGAGGGTAGTCC |
| *DYRK1A* | CCAAACATAAGTGACCAACAGG | TGTTGGTGTCTTCGCTTCTT |
| *GLI2* | GAGCACTACCTCCGTTCTGT | CGAGGGTCATCTGGTGGTAA |
| *PSMC4* | CGCCGCCAGAAGAGATTG | TTCACGGACAGCCAACATTC |
| *MYSM1* | CAGTACGTCCTGGCCTGAA | GCTTCTACTGCATCTTTTCTGTC |
| *DHRS4* | AGGCCTCTGTGCCCGGGCTTGGAAT | TGCTGCTTCCGGCTGCTGACGACCA |
| *MCM7* | AGGATGATGAACTCGGGAAG | TGTACGGCATCAGCAAAGAG |
| *HTR7* | GGGACCTGAGGACCACCTAT | TAGCACCCACACAGATACCG |
| *PLD6* | CGTCACCGACTGCGACTAC | CCAGTGATGAGCACCCTCTT |
| *EFNB1* | AAGAACCTGGAGCCCGTATC | GGGTCGAGAACTGTGCTACA |
| *EPHA4* | TTTGTCATCAGCCGGAGACG | CTCTCGCACTGCTTGGTTGG |
| *ROCK1* | CGGAAGTGAATTCGGATTGT | TCCAAATGCACCTCTACCAA |
| *EP300* | CACTGTGCATCTTCTCGACA | CTAACAGTGCTTAGGTTGGGG |
| *CYP26A1* | TTTGGAGGACACGAAACCAC | GGTCTCCTTAATAACACACCCG |
| *MAPK10* | TGGAAGTGGGAGACTCAACC | TTGGTTCTGAAAGGGTCTGC |
| *TRIM24* | GTTGGAGTCATTCGTTGCCC | TGCAGAGCCATTCAACACAC |
| *RARβ* | AGTGAGTCCTGGGCAAATCC | GGGTTTGTACACTCGAGGGG |
| *NESTIN* | CAGCGTTGGAACAGAGGTTGG | TGGCACAGGTGTCTCAAGGGTAG |
| *MESP1* | GTGCTGGCTCTGTTGGAGA | CAGAGACGGCGTCAGTTGT |
